# Effect of Kombucha Exposure on Corrosion Resistance of MIM Orthodontic Brackets: Geometry–Electrochemistry Coupling and Oral Health Implications (MIM-316L vs. Commercial)

**DOI:** 10.3390/ma19020400

**Published:** 2026-01-19

**Authors:** Anna Ziębowicz, Wiktoria Groelich, Klaudiusz Gołombek, Karolina Wilk

**Affiliations:** 1 Department of Biomaterials and Medical Devices Engineering, Silesian University of Technology, 41-800 Zabrze, Poland; wg301436@student.polsl.pl (W.G.); karolina.wilk@polsl.pl (K.W.); 2 Materials Testing Laboratory, Silesian University of Technology, 44-100 Gliwice, Poland; klaudiusz.golombek@polsl.pl

**Keywords:** MIM, orthodontic brackets, 316L, kombucha, roughness, wettability, pitting corrosion, SEM, energy-dispersive X-ray spectroscopy (EDS), oral health

## Abstract

Metal Injection Molding (MIM) enables complex orthodontic-bracket geometries but can introduce surface and geometric discontinuities that act as initiation sites for crevice and pitting corrosion. The effect of acidic, kombucha-like exposure on corrosion and repassivation was assessed for MIM-316L brackets relative to a commercial comparator, and the coupling between surface quality (roughness and wettability) and localized damage at scanning electron microscopy (SEM)-identified hot-spots was examined. Kombucha was characterized by pH and titratable acidity. Surfaces were characterized by SEM, areal roughness metrics (*R_a*, *S_a*, *S_z*, and *A2*), and wettability by sessile-drop goniometry. Electrochemical behavior in artificial saliva was measured using open-circuit potential and cyclic potentiodynamic polarization (ASTM F2129/G59), and a qualitative magnetic check was included as a pragmatic quality-assurance screen. Exposure in kombucha reduced breakdown and repassivation potentials and increased passive current density, with the strongest effects co-localizing geometric discontinuities. Commercial brackets exhibited markedly poorer surface quality (notably higher *S_z*), amplifying acidity-driven susceptibility. These findings indicate that, under acidic challenges, surface/geometry quality dominates corrosion behavior; non-magnetic-phase compliance and simple chairside screening (e.g., magnet test), alongside tighter manufacturing controls on roughness and edge finish, should be incorporated into clinical and industrial quality assurance (QA).

## 1. Introduction

Metal orthodontic brackets, typically manufactured from stainless steel, despite the availability of esthetic alternatives (ceramic, polymeric, and aligners), remain a mainstay of comprehensive treatment, owing to cost-effectiveness, mechanical robustness, and precise control of tooth movement with standardized slots and auxiliaries [1]. Their widespread use in fixed, multibracket appliances focuses attention on the as-manufactured surface state; in a warm, humid, microbially active oral environment, microdefects become privileged sites for electrolyte retention, oxygen depletion, and biofilm colonization, all of which facilitate the onset of corrosion [2,3].

Metal Injection Molding (MIM) enables single-piece brackets with complex features (tie wings, hooks, and slot geometry) and consistent dimensions across large batches [4]. The route, however, can leave microporosity, microcrevices, and sharp edges after de-binding/sintering and post-processing. These discontinuities concentrate mechanical and electrochemical stresses, locally perturbing the passive film and altering the balance between passivation and dissolution [5,6]. Evidence from surface-sensitive studies indicates that surface quality—captured by roughness descriptors such as *R_a*/*S_a* (average) and *S_z* (peak-to-valley amplitude), as well as edge finish—plays a primary role in the initiation of crevice/pitting events and the kinetics of repassivation in oral electrolytes [7,8]. In parallel, wettability governs liquid infiltration into features; lower contact angles promote capillary penetration and longer residence times of acidic fluids within recesses, thereby strengthening the geometry–electrochemistry coupling [9,10]. Wettability is also influenced by surface topography: increasing topographic complexity/roughness can reduce apparent wettability and intensify contact-line pinning (contact angle hysteresis), which in turn affects droplet spreading and retention on the surface [11].

Dietary acids compound these mechanisms. Beverages frequently consumed during orthodontic treatment lower interfacial pH and buffering capacity, accelerating depassivation of stainless-steel components and modifying electrochemical parameters (e.g., ↓E_p/E_rp, ↑*i_pass*) [12,13]. 

Recent studies have specifically examined corrosion and ion release of orthodontic stainless steels/brackets under exposure to acidic beverages and oral electrolytes, highlighting the sensitivity of electrochemical parameters to pH, organic acids, and surface finishing [14]. Other reports emphasized that manufacturing-related surface heterogeneities (edges, pores, and microcrevices) can act as initiation sites for localized attack and biofilm retention, motivating combined surface–electrochemistry workflows [15,16]. However, fermented beverages such as kombucha—characterized by high titratable acidity and batch-to-batch variability—remain comparatively underexplored in this context, particularly in direct comparisons between MIM and conventionally manufactured brackets. Kombucha, a fermented drink rich in organic acids, is a realistic yet understudied exposure; its pH and titratable acidity depend on fermentation conditions and recipe and often fall within 2.5–3.5, making it a stringent challenge for passive films [17,18,19]. Additionally, during the fermentation process of kombucha, newly emerging bacterial and yeast strains contribute to the development of a symbiotic culture of bacteria and yeast (SCOBY), which produces an organic biofilm. This biofilm may potentially adhere to dental and gingival surfaces during consumption, raising concerns regarding oral hygiene and the interaction with metallic elements [18]. Clinically common sipping patterns extend exposure time and increase the likelihood that acids infiltrate geometric hot-spots, especially on rough or poorly finished surfaces [13,17,19].

These processes are relevant not only to material degradation but also to stomatopathies, including mucosal irritation and hypersensitivity phenomena associated with corrosion products, and to plaque retention in bracket recesses that complicate hygiene [20]. Consequently, quality assurance (QA) should weigh surface topography and finish at least as heavily as nominal alloy choice. A practical implication from our preliminary observations is the usefulness of a simple chairside magnet test: incidental magnetic response in certain commercial brackets can flag batches for further verification and aligns with efforts to minimize susceptibility-related effects (e.g., MRI artifacts) in dental appliances [21].

Unlike many previous studies that examined orthodontic metals primarily under generic acidic beverages, short or simplified exposure protocols, or within a single material/manufacturing context, our work targets a clinically plausible but underexplored challenge: repeated exposure to kombucha, a fermented drink with high titratable acidity and batch-dependent composition. To address this gap, we applied a standardized 15-day kombucha pre-conditioning protocol designed to emulate regular consumption and evaluated brackets in the as-received and post-exposure states, as well as post-test after electrochemical measurements. We combined electrochemical assessment with SEM characterization to identify localized corrosion features at geometrical hot-spots, and we complemented these observations with quantitative surface descriptors (areal roughness and wettability). By directly comparing MIM 316L brackets with a commercial bracket material and anchoring corrosion outcomes to measurable surface features (rather than relying on correlation-based inference), we aim to provide actionable guidance for manufacturing quality assurance and clinically relevant risk awareness.

## 2. Materials and Methods

### 2.1. Samples and Media

Brackets. Test specimens comprised the following samples: “MIM-316L” (single supplier Experteam Sp. z. o. o., Białystok, Poland; one manufacturing lot) and a commercial bracket obtained as-received (no surface finishing beyond the vendor’s routine)—“CB-ASR”. Samples of 316L stainless steel manufactured using the MIM technique were selected as reference materials, as the majority of commercially available metallic orthodontic brackets are produced by this method. Among the most widely utilized materials in the MIM process is 316L stainless steel, particularly used to produce components intended for orthodontic applications [22,23]. Among the advantages of MIM in the production of components that are both small and geometrically complex, manufacturers highlight the ability to fabricate a bracket (even with a hook) as a single, monolithic part (without joints), as well as the high dimensional accuracy achievable through this method [24,25,26,27].

Terminology. The initial state prior to kombucha exposure is referred to as the as-received state. Samples subjected to 15-day kombucha pre-conditioning are referred to as post-exposure, and specimens after electrochemical testing are referred to as post-test.

For MIM specimens, the term as-received refers to the baseline surface after the standardized preparation (applied consistently to all MIM samples).

Metallographic preparation and etching. Mounted cross-sections were ground to 220 grit and polished to 0.04 µm colloidal silica. Sections were etched in an ethanol–FeCl_3_–HCl etchant for stainless steel under fixed conditions (concentration, 21–23 °C, 15–30 s), selected for revealing the austenitic 316L microstructure. Because an atypical etch response was observed in the commercial as-received bracket under these conditions, we performed confirmatory SEM–EDS (Section 2.4) on the same sample set to verify the composition. All etching parameters were identical across materials; three non-overlapping fields were examined per specimen. Where electrochemical tests required a defined surface area, non-tested regions were masked with chemically resistant lacquer to yield an exposed area A (±0.5 mm^2^) measured by planimetry.

Media. A physiologically relevant artificial saliva (composition specified in Supplementary Methods) was prepared from analytical-grade reagents according to a standardized formulation (see Supplementary Methods; based on [28]).

**Figure 1:**
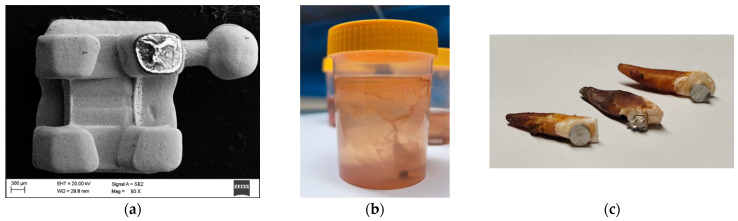
(**a**) As-received state of CB-ASR, (**b**) MIM-316L sample during exposure, and (**c**) extracted teeth with commercial bracket and MIM-316L samples attached, after exposure.

The artificial saliva contained NaCl (0.4 g/L), KCl (0.4 g/L), CaCl_2_·2H_2_O (0.795 g/L), NaH_2_PO_4_·2H_2_O (0.78 g/L), urea (1.0 g/L), and Na_2_S·9H_2_O (0.005 g/L), and was buffered to pH 6.8–7.0 at 37 °C. Na_2_S·9H_2_O was included in trace concentration as a sulfide source to better approximate oral-like, partially reducing conditions and sulfide-associated tarnish/corrosion phenomena, thereby providing a more clinically relevant electrolyte for assessing passive film stability and localized corrosion susceptibility. To replicate real-world conditions, i.e., regular kombucha consumption by a patient treated with fixed orthodontic appliances, the samples were placed in the incubator for 15 days at a temperature of 37 °C, which simulated the oral environment (kombucha was used as a pre-conditioning agent). The incubator contained not only the research material but also extracted teeth with the samples attached (in accordance with clinical procedures). Kombucha variants were produced or sourced with documented fermentation time/temperature and sugar loading; each batch was characterized by pH and titratable acidity (TTA) using standard potentiometry and acid–base titration protocols [17,18,19]. Prior to testing, all media were filtered (0.22 µm) and equilibrated at 37 °C; dissolved oxygen and conductivity were logged to account for aeration effects. The elements placed in the kombucha are shown in Figure 1.

Prior to immersion, the kombucha had an initial pH of 3.6 and an acetic acid content slightly below 1%. After 15 days of exposure to the tested materials, the solution became slightly more acidic (pH = 3.51), and the acetic acid concentration increased. Kombucha batches were monitored using potentiometric pH measurements and titratable acidity (TTA) determined by acid–base titration. Batch-to-batch variability was recorded to reflect realistic consumer exposure.

### 2.2. Surface and Wettability Characterization

Roughness (areal). R_a_ denotes the arithmetic mean roughness of a 2D line profile, whereas S_a_ denotes the arithmetic mean height of a 3D areal dataset measured over a surface patch. As bracket surfaces are spatially heterogeneous, areal parameters (S_a_ and S_z) are treated as primary descriptors in accordance with ISO 25178-2 [29]; R_a_ is reported only for comparability with the legacy literature, where applicable. Areal texture was quantified by confocal profilometry on VK-X3000 (Keyence, Keyence Corporation, Osaka, Japan) per ISO 25178-2 [29]. For confocal scans, fields of view (FOVs) of 500–1000 µm with lateral sampling ≤ 1 µm and vertical resolution ≤ 10 nm were acquired in ≥ 5 regions of interest (ROIs) per bracket (slot floor, tie-wing filets, and external edge). Prior to parameterization, form removal and scale-limited filtering followed ISO recommendations (Gaussian/S-L filtering; cut-offs explicitly stated in the Results section). Reported metrics included *R_a*/*S_a* (arithmetic mean height), *S_z* (maximum height, peak-to-valley amplitude), and *A2* (areal amplitude-related parameter), each averaged across ROIs. Atomic force microscopy (AFM, tapping mode, silicon probe, and nominal tip radius < 10 nm) was used in smaller FOVs (e.g., 20–50 µm) to validate sub-micron features detected by confocal imaging. For both modalities, outlier rejection was restricted to obvious artifacts (dust/particle spikes) identified by robust thresholding; raw datasets remain in the instrument’s proprietary format and were used to extract roughness parameters.

Wettability. Static contact angles were measured by sessile-drop goniometry per ASTM D7334 [30] (Attension^®^ Theta, Biolin Scientific, Gothenburg, Sweden). Drops of 2.0 ± 0.2 µL (DI water) were dispensed at 0.5–1.0 µL s^−1^; the contact angle was recorded by the instrument within 5 s of deposition. Temperature was maintained at 23 ± 1 °C and 50 ± 5% RH. Each value reflects the mean of ≥5 droplets placed on distinct ROIs mirroring the roughness sampling scheme. Prior to measurements, specimens were rinsed in DI water, dried with nitrogen, and rested for 30 min to minimize residual solvent effects. Outliers due to pinning on macroscopic defects were annotated rather than discarded.

SEM hot-spot mapping. SEM (secondary/SE and backscatter/BSE, 5–15 kV) was used to identify geometry-induced hot-spots (slot corners, tie-wing/slot intersections, and edge burrs) (FE-SEM, Supra 35, Carl Zeiss, Oberkochen, Germany). Each ROI was imaged at low (100–200×) and high (3000–8000×) magnification. A standardized defect taxonomy (pits, micro-crevices, incomplete fills, and sharp-edge radii) supported semi-quantitative counts per ROI, supported semi-quantitative comparisons/interpretation. Where charging risk existed, a thin conductive path (carbon paint away from the ROI) was applied without covering the analysis area.

Microhardness. Examination of the commercial bracket was conducted using Vickers method, with an applied load 10 N on a DuraScan Microhardness Tester (Struers A/S, Ballerup, Denmark).

### 2.3. Electrochemical Testing

Measurements were performed in a three-electrode cell at 37 ± 0.5 °C with the specimen as the working electrode, Ag/AgCl (3 M KCl) as the reference, and platinum as the counter electrode. Artificial saliva served as the electrolyte for all electrochemical tests. Prior to polarization, the open-circuit potential (OCP) was monitored for ≥30 min or until drift fell below 1 mV·min^−1^. Cyclic potentiodynamic polarization was performed following ASTM F2129/G59 [28] at 0.5–1.0 mV·s^−1^, from −250 mV vs. OCP to a predetermined upper limit or passivity breakdown, followed by a reverse scan (VoltaLab PGP201, Radiometer Analytical, Villeurbanne, France). Breakdown potential (*E_p*), repassivation potential (*E_rp*), and mean passive current density (*i_pass*) were extracted using standard definitions [29,30]. Currents were normalized by the exposed area (*A*, A·cm^−2^). Replicates (n ≥ 3 per condition) were tested in randomized order.

### 2.4. Microstructure and Chemical Composition

SEM/chemical composition (EDS). Energy-dispersive X-ray spectroscopy was performed in the SEM (15–20 kV; dwell ≥ 50 ms/pixel) on representative areas and hot-spots (FE-SEM, Supra 35, Carl Zeiss, Oberkochen, Germany and EDS, Trident XM4 series, Oxford Instruments, High Wycombe, UK). Spectra were acquired in triplicate per ROI, background-corrected, and quantified using ZAF corrections. Calibration was verified on certified 316L references. Acquisition parameters (accelerating voltage, live time, and working distance) were held constantly across groups.

## 3. Results

### 3.1. Samples and Media

The kombucha was analyzed for acetic acid content and pH. The initial CH_3_COOH level was consistent with the literature data, at approximately 1% of the beverage [31]. After incubation, titratable acidity (reported as acetic acid equivalents) increased and pH decreased. One post-exposure TTA/acid-content measurement yielded an implausibly high value relative to typical kombucha compositions; because it was a single, non-reproducible result and may reflect an analytical/handling artifact (e.g., endpoint detection or evaporation), it was treated as an outlier and excluded from mechanistic interpretation. This observation is reported transparently and will be validated in follow-up measurements with tighter evaporation control and titration standardization. Conclusions are therefore based on representative batches.

**Figure 2:**
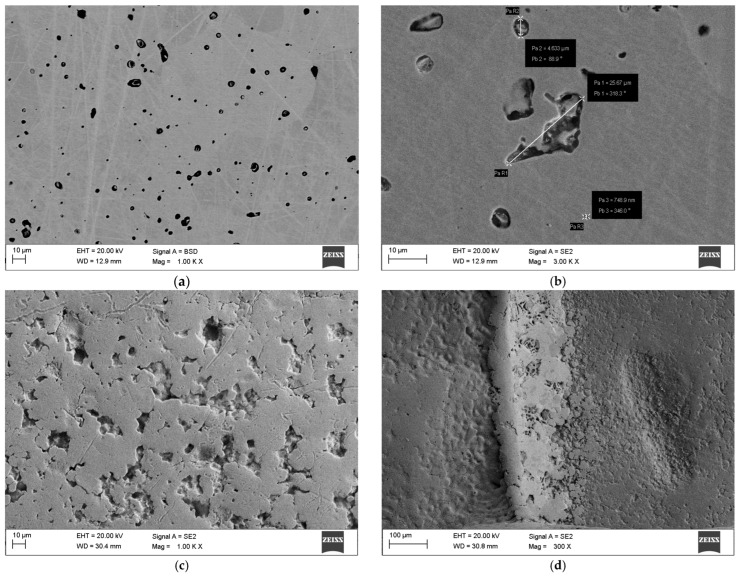
Co-registration of SEM hot-spots (as-received state). (**a**,**b**) MIM-316L, as-received—representative ROIs at the slot floor and tie-wing filet showing low defect density and smooth edge transitions. (**c**,**d**) Commercial bracket (CB-ASR), as-receivedsharp-edge radii, micro-pits, and incomplete fills at the slot corner and outer edge.

In addition, teeth exposed to the liquid turned dark brown, suggesting a potential staining risk during frequent or prolonged consumption of fermented beverages. Rinsing the mouth with water may be a simple preventive measure to reduce residual acids and chromogenic compounds. Macroscopic inspection of the orthodontic brackets also revealed a shape/surface defect (Figure 2c,d), which may act as a local site for electrolyte retention and increase susceptibility to pitting corrosion. The presence of such defects may reflect variability in manufacturing quality and supports the need for quality control screening.

### 3.2. Surface and Wettability Examinations 

MIM specimens exhibited order-of-magnitude lower roughness than the commercial bracket (as-received): *R_a* = 0.02 ± 0.01 µm; *S_a* = 0.02 ± 0.01 µm (MIM) versus *R_a* = 0.14 ± 0.08 µm; and *S_a* = 0.05 ± 0.08 µm (commercial). Across all conditions, *S_a* remained lower for MIM than for the commercial bracket, both as-received and post-exposure in kombucha and subsequent electrochemical testing in artificial saliva. Peak-to-valley amplitude (*S_z*) for the commercial bracket was 4.03 ± 0.77 µm in the as-received state and 4.49 ± 0.74 µm after kombucha pre-exposure plus corrosion testing, while the highest *S_z* was observed in the commercial bracket subjected only to the electrochemical test (*S_z* = 12.38 ± 2.52 µm), indicating extreme surface discontinuities at geometric hot-spots. 

The markedly lower *R_a*/*S_a* of MIM-316L specimens reflects their metallographic preparation (protocolled grinding/polishing) and does not represent a typical “as-received” finish. We therefore interpret roughness-driven susceptibility primarily through commercial as-received topography—especially the recorded *S_z* = 12.38 ± 2.52 µm—as a clinically relevant risk factor for pit/crevice initiation at bracket hot-spots. Future comparisons should include MIM-316L “as-sintered” and commercial as-received with matched post-processing (e.g., electropolishing/passivation) to decouple process vs. finish effects.

Wettability. The as-received MIM-316L surfaces were hydrophilic (*θ* = 51.28 ± 7.58°), and kombucha pre-exposure decreased *θ* to 46.56 ± 4.09° (Δ*θ* ≈ −4.7°; *n* = 5; *p* = 0.574/95% CI [40.05°, 53.07°]). Given *θ* < 90° in both states, this modest shift is consistent with a Wenzel-type amplification of hydrophilicity by increased roughness and/or with surface chemistry changes induced by the acidic medium. We therefore interpret the wettability change as a combined topography–chemistry effect; attribution to roughness alone would require complementary surface chemistry data (e.g., X-ray Photoelectron Spectroscopy XPS/Time-of-Flight Secondary Ion Mass Spectrometry ToF-SIMS). Clinically, lower θ may enhance capillary infiltration and the residence of acidic fluids at bracket hot-spots.

SEM hot-spot mapping. Scanning electron microscopy (SEM) observations of the tested elements in their as-received state revealed that the surface quality of commercial orthodontic brackets was notably poor. Numerous pores and craters provided ideal sites for the initiation of pitting corrosion. Representative surface discontinuities identified and measured for the orthodontic bracket and MIM-316L samples are shown in Figure 2. Compared to the brackets, the surface of MIM-316L samples exhibited fewer pores, which may be attributed to their simpler geometry. The high number of surface irregularities observed on CB-ASR brackets may result from an improperly conducted sintering process, which constitutes the final stage of the MIM technology.

**Figure 3:**
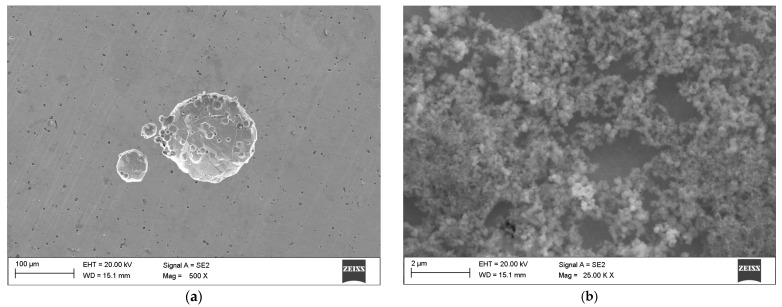

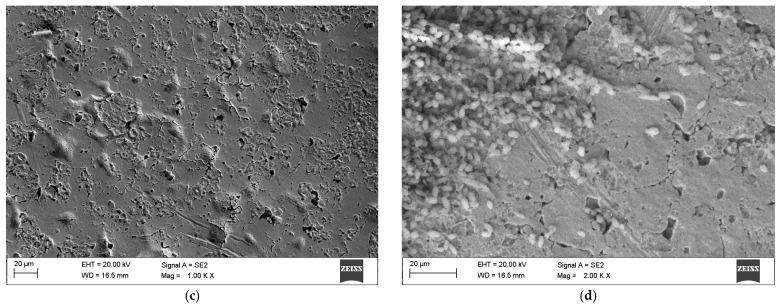
Co-registration of SEM hot-spots after exposure to kombucha (37 °C, 15 days/after kombucha preconditioning). (**a**,**b**) MIM-316L, post-exposure—limited topographical change; isolated shallow pits at the tie-wing/slot intersection. (**c**,**d**) CB-ASR, post-exposure—increased pit density and crack-like features at geometric discontinuities; a filamentous surface film consistent with biofilm is visible.

Exposure of the samples to simulated conditions mimicking regular kombucha consumption (elevated temperature and acidic environment) led to the formation of a biofilm on their surfaces (Figure 3), indicating probable colonization of the metallic surface by the bacteria and fungi present in kombucha. The biofilm exhibited a web-like or fibrous morphology, wrapping around the orthodontic bracket. Pitting corrosion resistance tests conducted on the samples in their as-received state further deteriorated their surface condition. Pre-existing pores on the CB-ASR surface were deepened, likely due to current flow through the samples. 

**Figure 4:**
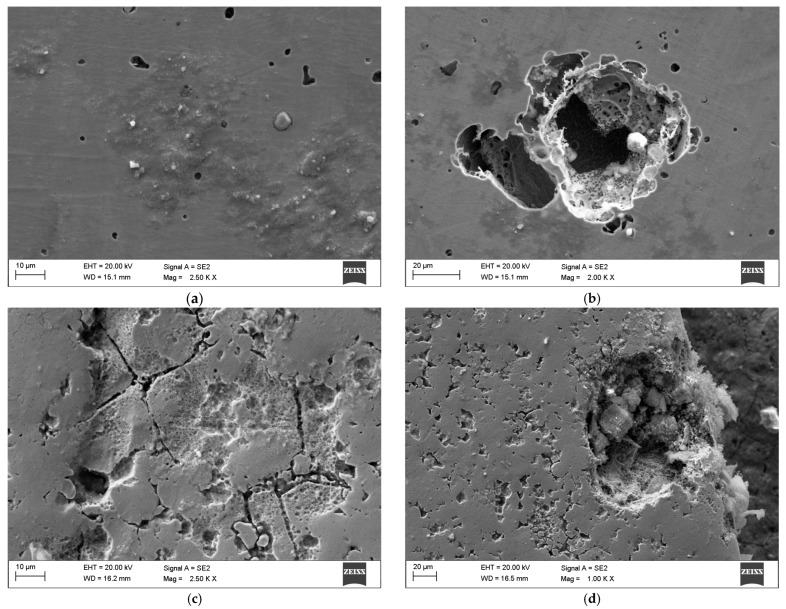
Co-registration of SEM hot-spots after cyclic potentiodynamic polarization (artificial saliva). (**a**,**b**) MIM-316L, post-test—discrete, shallow pits at pre-identified hot-spots; surrounding passive film largely intact. (**c**,**d**) CB-ASR, post-test—wider/deeper pits and micro-crevice networks aligned with sharp edge transitions; evidence of pit coalescence.

**Figure 5:**
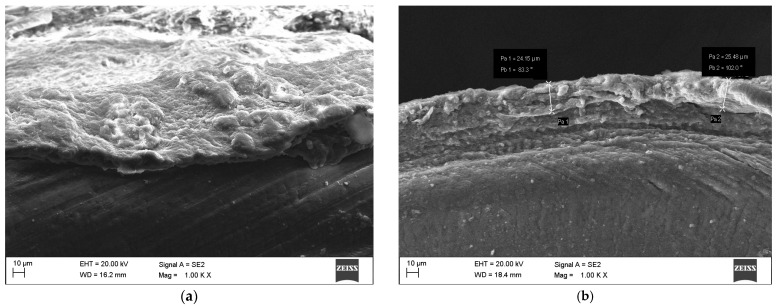
Extracted tooth carrying CB-ASR after kombucha exposure: (**a**) cross-section with biofilm topography; (**b**) cross-section with measurement points indicating biofilm thickness.

Pitting corrosion pits were observed in both tested variants. The CB-ASR samples exhibited a greater number of pits, and their overall surface showed more cracks and irregularities compared to MIM-316L. However, the corrosion pits observed in the CB-ASR elements were more extensive (Figure 4). A distinct presence of biofilm was also noted on the surface of teeth immersed in kombucha, with an estimated thickness of approximately 25 μm (Figure 5).

### 3.3. Electrochemical Testing

Breakdown and repassivation. In artificial saliva at 37 °C (Ag/AgCl, 3 M KCl; scan 0.5–1.0 mV s^−1^), the MIM samples exhibited a mean breakdown potential (Ep) of 1.14 ± 0.18 V before and 1.24 ± 0.02 V after 15-day kombucha pre-exposure. In contrast, the commercial bracket (as-received) showed a >0.5 V decrease in *E_p* upon pre-exposure (from 1.36 V to 0.83 V), indicating increased susceptibility to localized breakdown. Repassivation potentials, *E_rp*, for MIM were 0.07 ± 0.11 V (before) and 0.11 ± 0.15 V (after), consistently higher than for the commercial bracket (0.01 V before; −0.17 V after), yielding wider hysteresis for the latter.

Polarization resistance and passive current. The polarization resistance (R_p, Ω·cm^2^) for MIM was 843 ± 402 in the as-received state and decreased after pre-exposure, whereas the commercial bracket exhibited the lowest R_p across all conditions (as-received ≈208, post-exposure further reduced). Passive current densities, i_pass, increased after kombucha pre-exposure in both cohorts, with larger increments for the commercial bracket.

**Figure 6:**
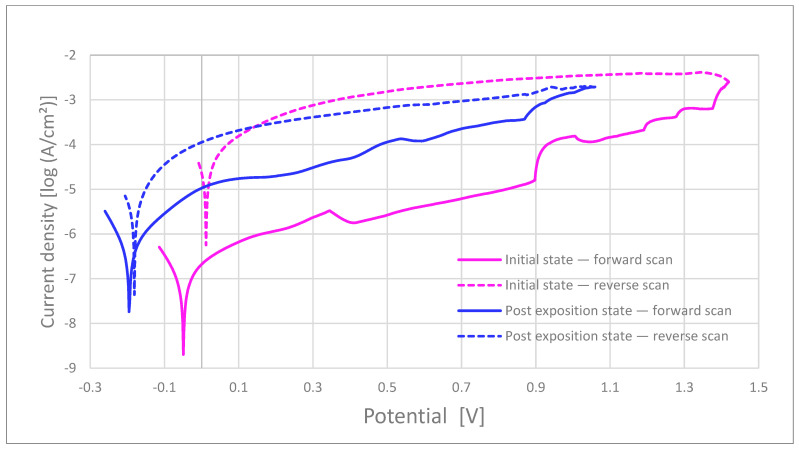
Representative polarization curves for commercial bracket (CB-ASR). Solid lines: forward scan; dashed lines: reverse scan.

Morphology–electrochemistry coupling. Post-test 3D profilometry/SEM confirmed a higher pit density and greater peak-to-valley amplitude (S_z) after pre-exposure; these metrics co-localized with geometric hot-spots and were observed alongside with electrochemical susceptibility. Representative polarization curves for the commercial bracket (before/after) are shown in Figure 6. Commercial as-received brackets show broader hysteresis, indicating a greater pitting–repassivation gap.

### 3.4. Microstructure and Chemical Composition

**Figure 7:**
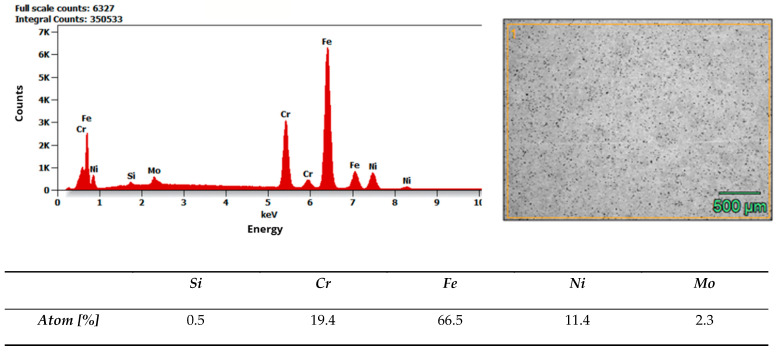
Results of EDS analysis of MIM-316L as-received state.

**Figure 8:**
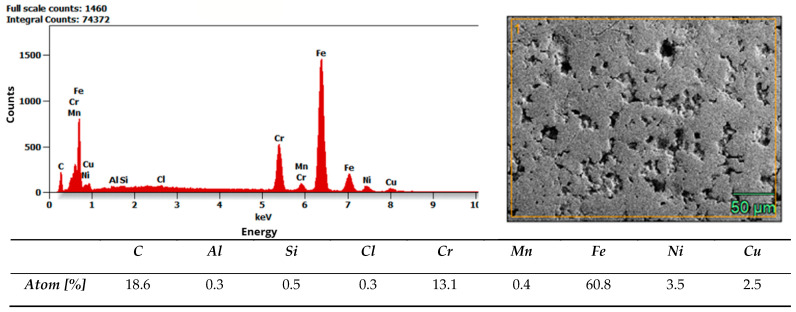
Results of EDS analysis of commercial bracket as-received.

Microstructure and composition. MIM-316L references showed uniform austenitic contrast after etching in ferric chloride–hydrochloric acid–ethanol etchant (100 ml C_2_H_5_OH, 3 g FeCl_3_, 1,5 ml HCl), consistent with the data sheet literature for 316L [32]. In contrast, the commercial as-received bracket exhibited irregular over-etching and poor phase delineation under identical conditions. SEM–EDS analysis acquired from multiple regions of interest (n ≥ 3 per field) did not detect Mo above the instrument’s limit of detection, while Fe–Cr–Ni peaks were present. Together with the incidental magnetic response and higher hardness of the commercial bracket (268 ± 12.28 HV10, when microhardness declared in data sheet of MIM-316L is 120 HV10), these findings indicate that the as-received comparator is Mo-free and off-spec relative to 316L, explaining its divergent etch behavior. Representative spectra and raw etch images are presented in Figure 7 and Figure 8. EDS analysis confirmed that the chemical composition of the MIM 316L samples was consistent with the specifications provided in the manufacturer’s data sheet [32]. In contrast, examination of the commercial orthodontic brackets revealed no detectable molybdenum content in the material—Figure 8. This could suggest that the brackets could not have been manufactured from austenitic stainless steel, specifically of the Cr-Ni-Mo type (e.g., 316L). However, to reliably confirm both the chemical composition and the phase constitution, XPS and X-ray Diffraction (XRD) analyses would be required. The detected chlorine may originate from the kombucha, a plant-derived, tea-based medium, as chloride ions naturally occur in plant tissues due to their essential physiological functions. It may also come from the artificial saliva solution used in the corrosion tests. In addition, trace amounts of chlorine can be associated with chloride-containing corrosion products formed on the metal surface.

## 4. Discussion

Pre-exposure to an acidic medium (kombucha) reduced corrosion resistance in all groups subsequently tested in artificial saliva. Breakdown and repassivation potentials (E_p/E_rp) decreased, while the passive current density (i_pass) increased. The strongest effects were observed at geometric discontinuities (“hot-spots”) identified by SEM. Compared with metallographically prepared MIM references, commercial brackets showed poorer surface quality (higher S_z, sharper edges, and more pronounced porosity). These features promote electrolyte retention, local depassivation, and pitting. Within the detection limits of EDS, Mo was not detected in the commercial brackets, whereas the MIM-316L references matched the supplier’s data sheet [32]. This compositional difference is consistent with the higher susceptibility of commercial brackets to localized corrosion. Intermittent magnetic attraction observed in some commercial brackets may serve as a pragmatic QA signal that warrants batch verification.

The discrepant etch response of the commercial bracket is unlikely to be explained by polishing or reagent variability, because the etching conditions were controlled and reproduced across three fields per specimen. Instead, the observations are consistent with composition and microstructural indicators. The absence of Mo by EDS, together with magnetic response (and, if measured, higher hardness), helps explain why the C_2_H_5_OH–FeCl_3_–HCl etchant—optimized for Mo-bearing austenitic 316L—did not yield the expected microstructural contrast. From a corrosion perspective, the lack of Mo also aligns with the lower localized corrosion resistance measured electrochemically relative to MIM-316L.

Overall, the results support the view that surface topography and edge finish—rather than the nominal alloy label alone—govern pit initiation and repassivation in oral electrolytes. High S_z and sharp geometric transitions amplify local concentration and stress gradients and lower the barrier to passive film rupture. Wettability can further increase risk: lower contact angles facilitate capillary infiltration of acidic electrolyte into crevices and prolong fluid residence in micro-recesses [9,10]. Kombucha pre-exposure (pH ~2.5–3.5; organic acids) may alter the surface state (e.g., adsorption of fermentation products and local cleaning/degreasing effects), which can contribute to poorer electrochemical metrics in subsequent artificial saliva tests [12,13,16,17,18,19]. The absence of Mo in commercial bracket matrices may further reduce pitting resistance compared with 316L, as Mo is known to strengthen passivity and suppress i_pass in the presence of aggressive anions [7,8].

Prior studies have linked roughness—including areal parameters defined in ISO 25178-2—to localized corrosion susceptibility in 3xx stainless steels, and have shown that wettability can modulate mass transport and degradation kinetics. Our observations extend this evidence to a dietary exposure scenario. Kombucha pre-exposure has rarely been explored in orthodontics and is more often discussed in the context of enamel erosion or bracket bond strength. Here, we apply a structured “kombucha → artificial saliva” protocol and combine an SEM-based hot-spot assessment with quantitative surface descriptors (R_a, S_a, S_z, and A2), wettability (contact angle), and electrochemical outcomes (E_p/E_rp, i_pass, and R_p).

Clinically, brackets with poorer finishing (high S_z and sharp edges) combined with acidic beverage exposure may create conditions conducive to local irritation/hypersensitivity reactions and increased biofilm retention. We therefore recommend a dual approach:

- Patient education (limit contact time with acidic drinks; rinse with water after intake);
- Quality control in manufacturing and clinics (define acceptance thresholds for critical regions; enforce edge-finish controls; verify Mo compliance where brackets are marketed as 316L; and use a rapid magnet check as a chairside warning prompt for further verification).

The environments were intentionally separated: kombucha served as pre-conditioning, followed by electrochemical measurements in artificial saliva. This design reflects a realistic pattern in which patients intermittently sip acidic drinks between periods dominated by saliva. The wider electrochemical hysteresis observed for commercial as-received brackets indicates a larger window in which pits can propagate before repassivation.

Limitations. Reference MIM specimens were metallographic mounts; therefore, their very low R_a/S_a values may not reflect the typical as-received finish of clinical brackets. EDS is qualitative/semi-quantitative and does not provide phase identification; although Mo was not detected in the commercial brackets across multiple ROIs, confirmatory phase/chemistry analyses (e.g., XRD/EBSD and/or surface-sensitive methods) were outside the scope of this study. One anomalous post-exposure TTA/pH event requires validation (e.g., evaporation control and replication, titration standardization). The set of commercial brackets (brands/batches) was limited. No surface-sensitive chemical characterization (e.g., XPS or ToF-SIMS) was performed; therefore, any discussion invoking chemical modification of the outermost surface remains speculative and should be tested in future work.

Future directions. Priorities include the following:

- A cross-brand audit (≥5 manufacturers; n ≥ 10/brand) to define *S_z*/*R_a* acceptance thresholds for critical regions;
- Composition/phase verification for commercial brackets (quantitative EDS and XRD/EBSD, and, where relevant, surface-sensitive chemistry);
- Long-term tests incorporating biofilm and pH cycling to better emulate clinical loading.

## 5. Conclusions

Within the limitations of this study, the following conclusions can be drawn. These conclusions refer to the as-received surface condition and clinically relevant finishing of the tested brackets, rather than the intrinsic superiority of a nominal alloy grade.

1. As-received geometry and surface finish are primary drivers. Complex components, such as orthodontic brackets, geometric discontinuities (sharp transitions and porosity/crevices), and high peak-to-valley amplitudes (S_z) in the as-received condition, promote localized electrolyte retention and facilitate the pit initiation observed by SEM.

2. Acid pre-conditioning worsens electrochemical metrics. Fifteen-day kombucha pre-conditioning at 37 °C reduced corrosion performance in subsequent artificial saliva tests across all groups (↓E_p/E_rp and ↑i_pass), with the lowest R_p observed for commercial as-received brackets.

3. As-received composition/quality control matters. Within the detection limits of SEM–EDS, Mo was not detected in some commercial as-received brackets, whereas the MIM-316L references matched the supplier’s data sheet; this difference may contribute to the higher susceptibility to localized corrosion observed in the tested condition. However, categorical alloy identification is beyond the scope of this study because EDS is semi-quantitative and no phase analysis (XRD/EBSD) was performed.

4. Actionable QA. The findings support quality assurance measures targeting as-received clinical performance, including surface-finish specifications in critical regions (e.g., S_z/R_a thresholds), edge-finish controls, and screening for Mo compliance where brackets are marketed as 316L. An incidental magnetic response may serve as a practical warning signal prompting batch verification.

5. Clinical guidance. Under acidic challenges, bracket surface features that favor fluid retention (roughness, sharp edges, and crevices) may increase the likelihood of localized degradation and plaque retention. Patients wearing fixed appliances should limit contact time with acidic beverages and rinse with water after intake.

6. Esthetic consideration. Darkening of teeth after kombucha incubation indicates a potential staining risk during frequent or prolonged exposure.

## Data Availability

The original contributions presented in this study are included in the article. Further inquiries can be directed to the corresponding author.

## References

[1] Fróis A., Santos A.C., Louro C.S. (2023). Corrosion of Fixed Orthodontic Appliances: Causes, Concerns, and Mitigation Strategies. Metals.

[2] Ziębowicz A., Woźniak A., Ziębowicz B., Gzik M., Tkacz E., Paszenda Z., Piętka E. (2017). The Influence of Technology on the Physicochemical and Electrochemical Properties of the Prosthetic Materials. Innovations in Biomedical Engineering.

[3] Ziębowicz A. (2023). ZrO_2_ Coating on Cobalt Alloys Substrate as a Factor to Minimize Denture Stomatitis.

[4] Zinelis S., Annousaki O., Makou M., Eliades T. (2005). Metallurgical Characterization of Orthodontic Brackets Produced by Metal Injection Molding (MIM). Angle Orthod..

[5] Espinoza-Montero P.J., Montero-Jiménez M., Fernández L., Paz J.L., Piñeiros J.L., Ceballos S.M. (2022). In Vitro Wearing Away of Orthodontic Brackets and Wires in Different Conditions: A Review. Heliyon.

[6] Doomen R.A., Nedeljkovic I., Kuitert R.B., Kleverlaan C.J., Aydin B. (2022). Corrosion of Orthodontic Brackets: Qualitative and Quantitative Surface Analysis. Angle Orthod..

[7] Tang Y., Dai N., Wu J., Jiang Y., Li J. (2019). Effect of Surface Roughness on Pitting Corrosion of 2205 Duplex Stainless Steel Investigated by Electrochemical Noise Measurements. Materials.

[8] Pereda M.D., Kang K.W., Bonetto R., Llorente C., Bilmes P., Gervasi C. (2012). Impact of Surface Treatment on the Corrosion Resistance of ASTM F138-F139 Stainless Steel for Biomedical Applications. Procedia Mater. Sci..

[9] Žemaitis A., Mimidis A., Papadopoulos A., Gečys P., Račiukaitis G., Stratakis E., Gedvilas M. (2020). Controlling the Wettability of Stainless Steel from Highly-Hydrophilic to Super-Hydrophobic by Femtosecond Laser-Induced Ripples and Nanospikes. RSC Adv..

[10] Estrada-Martínez J., Reyes-Gasga J., García-García R., Vargas-Becerril N., Zapata-Torres M.G., Gallardo-Rivas N.V., Mendoza-Martínez A.M., Paramo-García U. (2017). Wettability Modification of the AISI 304 and 316 Stainless Steel and Glass Surfaces by Titanium Oxide and Titanium Nitride Coating. Surf. Coat. Technol..

[11] (2026). Dynamic Wettability of Complex Fractal Isotropic Surfaces—Multiscale Correlations. Tribol. Int..

[12] Shahabi M., Jahanbin A., Esmaily H., Sharifi H., Salari S. (2011). Comparison of Some Dietary Habits on Corrosion Behavior of Stainless Steel Brackets: An in Vitro Study. J. Clin. Pediatr. Dent..

[13] Lee Y.-S., Yang P.-Y., Lee T.-H. (2022). The Effects of Beverage on Orthodontic Wires—Research Report. Taiwan. J. Orthod..

[14] Umurca D.G., Sasany R., Gülyurt M. (2025). Analysis of Corrosion, Surface Roughness, and Ion Release of Orthodontic Brackets in Simulated Gastric Acid and Saliva. Sci. Rep..

[15] Venkatesan K., Srinivasan B., Padmanabhan S. (2021). Adverse Effect of Consumption of Carbonated Soft Drinks on Orthodontic Treatment—A Systematic Review. Indian J. Dent. Res..

[16] Bougadi M.A.J., Reddy A., Biradar A., Telang V., Bharathi V.S., Panda A., Mehta M., Patil D. (2025). In Vitro Study on the Effect of Acidic Beverages on the Shear Bond Strength of Orthodontic Brackets. J. Pharm. Bioallied Sci..

[17] Bishop P., Pitts E.R., Budner D., WItrick K. (2022). Kombucha: Biochemical and Microbiological Impacts on the Chemical and Flavor Profile. Food Chem. Adv..

[18] de Oliveira P.V., da Silva Júnior A.H., de Oliveira C.R.S., Assumpção C.F., Ogeda C.H. (2023). Kombucha Benefits, Risks and Regulatory Frameworks: A Review. Food Chem. Adv..

[19] Cohen G., Sela D.A., Nolden A.A. (2023). Sucrose Concentration and Fermentation Temperature Impact the Sensory Characteristics and Liking of Kombucha. Foods.

[20] Iosif C., Cuc S., Prodan D., Moldovan M., Petean I., Badea M.E., Sava S., Tonea A., Chifor R. (2022). Effects of Acidic Environments on Dental Structures after Bracket Debonding. Int. J. Mol. Sci..

[21] Wang Z.J., Park Y.J., Morriss M.C., Seo Y., Nguyen T., Hallac R.R., Nava A., Chopra R., Chatzinoff Y., Price K. (2018). Correcting B0 Field Distortions in MRI Caused by Stainless Steel Orthodontic Appliances at 1.5 T Using Permanent Magnets—A Head Phantom Study. Sci. Rep..

[22] Lin C.-M., Wu J.-J., Tan C.-M. (2020). Processing Optimization for Metal Injection Molding of Orthodontic Braces Considering Powder Concentration Distribution of Feedstock. Polymers.

[23] Colmant M., Fawaz P., Stanton K., MacMichael O., Vande Vannet B. (2023). Microhardness and Chemical Composition of Different Metallic Brackets: An In Vitro Study. Dent. J..

[24] Dentaurum Discovery^®^ Smart—Modern Bracket System Made of Metal—Discovery_Smart. https://www.dentaurum.com/lp/eng/discovery-smart.aspx.

[25] (2024). Leone—Orthodontics Product Catalogue. https://leone.it/english/services/download/CAT-ORTHO-2024-ENG.pdf?.

[26] 3M Science Applied to Life. Victory Series^TM^—Superior Fit Buccal Tubes. https://docs.exhausmed.com/docs/3m/catalogue/cat_superior-fit-bucal-tube_3m_2015.pdf.

[27] Solutions for Nickel Allergies. Forestadent: Pforzheim, Germany. https://forestadent.com/en/products-solutions/solutions-for-nickel-allergies/.

[28] (2020). Dentistry—Corrosion Test Methods for Metallic Materials.

[29] (2021). Geometrical Product Specifications (GPS)—Surface Texture: Areal Part 2: Terms, Definitions and Surface Texture Parameters.

[30] (2022). Standard Practice for Surface Wettability of Coatings, Substrates and Pigments by Advancing Contact Angle Measurement.

[31] Greenwalt C.J., Ledford R.A., Steinkraus K. (1998). Determination and Characterization of the Antimicrobial Activity of the Fermented Tea*Kombucha*. LWT Food Sci. Technol..

[32] (2016). Catamold^®^ 316L—Technical Data Sheet. https://hpmltd.com/wp-content/uploads/2022/09/catamold-product-range.pdf.

